# Osteolytic vs. Osteoblastic Metastatic Lesion: Computational Modeling of the Mechanical Behavior in the Human Vertebra after Screws Fixation Procedure

**DOI:** 10.3390/jcm11102850

**Published:** 2022-05-18

**Authors:** Daniele Bianchi, Cristina Falcinelli, Leonardo Molinari, Alessio Gizzi, Alberto Di Martino

**Affiliations:** 1 Department of Engineering, University of Rome Campus Bio-Medico, 00128 Rome, Italy; d.bianchi@unicampus.it; 2 Department INGEO, University of Chieti-Pescara, 66100 Chieti, Italy; cristina.falcinelli@unich.it; 3 Department of Mathematics and Computer Science, Emory University, Atlanta, GA 30322, USA; leonardo.molinari@emory.edu; 4 Department of Biomedical and Neuromotor Science, University of Bologna, 40126 Bologna, Italy; dimartino.cbm@gmail.com; 5 1st Orthopaedic and Traumatologic Clinic, IRCCS Istituto Ortopedico Rizzoli, 40136 Bologna, Italy; 6 Sidney Kimmel Medical College, Thomas Jefferson University, Philadelphia, PA 19107, USA

**Keywords:** metastatic vertebra, lytic lesions, osteoblastic lesions, finite element analysis, Bonemetastasis interaction, constitutive modeling, fracture risk

## Abstract

Metastatic lesions compromise the mechanical integrity of vertebrae, increasing the fracture risk. Screw fixation is usually performed to guarantee spinal stability and prevent dramatic fracture events. Accordingly, predicting the overall mechanical response in such conditions is critical to planning and optimizing surgical treatment. This work proposes an image-based finite element computational approach describing the mechanical behavior of a patient-specific instrumented metastatic vertebra by assessing the effect of lesion size, location, type, and shape on the fracture load and fracture patterns under physiological loading conditions. A specific constitutive model for metastasis is integrated to account for the effect of the diseased tissue on the bone material properties. Computational results demonstrate that size, location, and type of metastasis significantly affect the overall vertebral mechanical response and suggest a better way to account for these parameters in estimating the fracture risk. Combining multiple osteolytic lesions to account for the irregular shape of the overall metastatic tissue does not significantly affect the vertebra fracture load. In addition, the combination of loading mode and metastasis type is shown for the first time as a critical modeling parameter in determining fracture risk. The proposed computational approach moves toward defining a clinically integrated tool to improve the management of metastatic vertebrae and quantitatively evaluate fracture risk.

## 1. Introduction

The vertebra is one of the most common sites that can be affected by metastasis [1,2], and nearly 5% to 10% of patients with tumors develop vertebral metastases [3]. The presence of vertebral metastasis can induce a vertebral fracture (10–30% of all cancer patients [4]) that causes pain, instability, limited mobility, and neurological alterations significantly affecting the quality of life [5]. A vertebral fracture is mainly related to the mechanical changes that metastasis induces in the vertebral environment. In particular, the presence of a tumor, which is characterized by different mechanical properties than bone, causes changes in bone material properties, leading to a significant alteration of bone mechanical strength and, thus, an increase in fracture occurrence probability. As such, evaluating fracture risk is essential to prevent critical events. In clinical practice, fracture risk assessment is still not so accurate. The clinical scoring systems [4] used to estimate a vertebral failure do not account for mechanical determinants of fracture (i.e., geometry, material properties, loading acting on the bone) that are key to evaluating its occurrence [6]. In the last decade, due to the need to improve the fracture risk assessment, mechanics-based computational tools such as image-based finite element (FE) modeling [7,8,9,10] were developed to better understand the mechanical behavior of vertebra with tumoral lesions. Campbell et al. [7] tested the ability of CT-based FE models of vertebrae in assessing bone fragility in patients suffering from multiple myeloma. They demonstrated that FE-derived vertebral compressive strength better classified patients with multiple myeloma who suffered from a fracture than densitometric or microstructural parameters. In the same context, Anitha et al. [8] demonstrated the need for advanced FE tools to predict individual fracture risk. It is essential to emphasize that neither study used a specific constitutive model for the tumoral lesion. Still, they derived the material properties using the same CT-based density–elasticity relationship used for a healthy bone.

Costa et al. [9] developed a subject-specific CT-based FE modeling approach to furnishing a comparative biomechanical assessment of vertebrae with lytic metastasis to the adjacent controls. They used the same constitutive law to model healthy tissue and lytic lesions. The authors did not find significant differences in ultimate force between vertebrae with lytic lesions and controls. However, lytic lesions were significantly weaker in several cases. Such a result suggests that patient-specific analyses are mandatory for better estimating the lesion’s effect on the vertebrae’s mechanical stability and thus for developing a personalized fracture risk index.

Stadelmann et al. [10] investigated the ability of standard FE methodologies to predict the strength of metastatic vertebral bodies for lytic, blastic, and mixed lesions. Their analysis showed a certain ability of the FE models to predict the overall vertebral strength compared to the experimental values. The authors used a standard BV/TV-driven constitutive law to build their FE simulations on healthy bone material constants. The negligible mass contribution of metastasis justified this assumption compared to the overall bone mass.

FE-based computational approaches have also been used to evaluate the impact of size and location of simulated lytic metastasis on the vertebra structural response, concluding that the effect of the size of the metastasis is more significant than that caused by the site of the lesion [11,12,13,14]. Whyne et al. [11] simulated osteolytic defects, modeling the tumoral tissue as a poroelastic isotropic material. Their simplified 2D model considered the aggregate modulus and hydraulic permeability varied to represent a spectrum of material properties found in lytic lesions, thus assigning specific material properties to the metastatic tissue. A hyperelastic material representation for the tumor tissue was used in the idealized 3D geometry studied in [12]. Recently, Galbusera et al. [13] included pseudo-spherical lytic lesions with random size and locations in a 3D CT-based FE computational model, assigning a different Young’s modulus (1 MPa) and Poisson’s ratio (0.45) to the tumor tissue. The simulated osteolytic lesions were modeled as holes in [14], not accounting for material properties that characterize the metastases and assuming that healthy tissue is not affected by the lesion. It is worth noting that the strategy of modeling the osteolytic defects as voids has also been used in experimental studies on metastatic vertebrae [15,16,17]. Finally, conversely to the osteolytic lesions, the osteoblastic lesions in the vertebra have not been extensively investigated using FE modeling except for the work of Stadelmann et al. [10] previously described.

The image-based FE modeling approaches developed so far have demonstrated the potential of computational modeling in furnishing a better understanding of the impact of metastatic disease on vertebra mechanical behavior. However, in most studies reported in the literature, metastasis was modeled as voids or by considering the same CT-based density–elasticity relationship used for healthy bone. Furthermore, any investigation concerning the presence of screw fixation was advanced in such a context. As such, the lack of specific constitutive modeling of metastasis and, more importantly, the inattention to possible effects that the metastatic lesion may have on the bone surrounding the lesion in the presence of fixation screws represent significant limitations of the modeling studies [5]. Developing models that account for the bone–metastasis interaction may be crucial to increasing the insight into the mechanical behavior of metastatic vertebra.

Bone cement augmentation procedures such as vertebroplasty or kyphoplasty are often used to treat patients with metastasis-related vertebral compression fractures or to restore vertebral body stability when mechanical integrity is significantly compromised by metastasis. However, it was reported that these procedures are not always effective [18,19]. In such cases, structural stabilization requires screw fixation [20,21] that consists of inserting different kinds of implants such as pedicle screws in the vertebra [20,22]. Following screw fixation, surgery complications can arise, e.g., construct failure, malposition of screws, neurologic deterioration, and deep and superficial wound infections [21,23,24]. Several retrospective studies have investigated complication rates after screw fixation for the treatment of spinal metastases, reporting between 15 and 47% of revisions. A higher complication rate (76%) was also found in the prospective study conducted by Dea et al. [23]. In the framework of surgical treatment planning to minimize the risk of complications for mechanical-based adverse events, it is crucial to evaluate the mechanical response of metastatic vertebra after screw fixation. Moreover, no studies in the literature investigate the mechanical behavior of instrumented metastatic vertebra.

The present work aims to evaluate in silico the mechanical behavior of instrumented metastatic vertebra through the development of an image-based FE computational tool. To this aim, we virtually insert the screws in the vertebra using the insertion trajectory that our previous analyses [25,26] showed as the most critical in terms of stress concentration. We adopted a modeling strategy for the metastatic tissue following the approach developed in [27,28]. Accordingly, we account for the effect of the metastatic tissue on the bone material properties reproducing bone–metastasis interaction. We then perform an extensive FE comparative analysis investigating the effect of different lesion types (i.e., osteolytic and osteoblastic). We also compare the lesion’s size, location, and shape in terms of fracture load and fracture patterns, considering the vertebra under two different loadings. A quantitative evaluation of stress- and strain-based failure criterion is further advanced, aiming to evaluate the influence of the adopted criterion on the comparative results, thus addressing the open problem concerning the optimal choice of the failure criterion. This study finally contributes to: (i) address the mechanical response of metastatic vertebra after screw fixation, (ii) account for a specific constitutive description of the metastatic tissue, and (iii) furnish a comprehensive overview of the influence of type, size, location, and shape of metastasis and failure criterion on the mechanical behavior of the vertebra.

## 2. Materials and Methods

### 2.1. Imaging and FE Geometric Reconstruction of the Screws-Vertebra Model

A CT spinal scan of a 49-year-old female patient was used in the present work. The following imaging parameters were set: 120 kVp, 489 mA, 0.8418 × 8418 mm pixel size, and 1 mm slice thickness. The images were acquired without a calibration phantom and were anonymized for their use, fulfilling the ethical protocols at the University Hospital Campus Bio-Medico of Rome.

**Figure 1:**
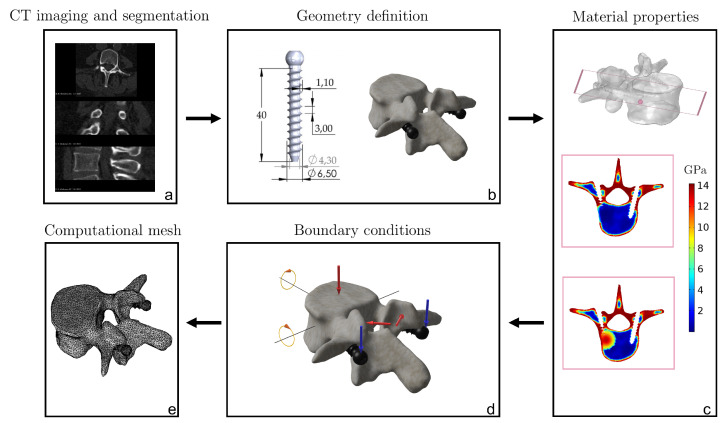
CT-based FE modeling procedure. Starting from CT scan (**a**), the vertebra geometry is reconstructed through segmentation (**b**). In addition, the CAD models of two pedicle screws are designed and virtually inserted in the vertebra (**b**). Then, a simulated metastasis with a spherical shape is included in the model, and it is assumed that the metastatic lesion induces an alteration of Young’s modulus in the bone region close to the metastasis (**c**). Appropriate boundary conditions are chosen to replicate realistic scenarios (**d**). Finally, the screws–vertebra model is discretized using ten-nodes tetrahedral elements (**e**).

In Figure 1 the main steps of the developed computational approach are shown: (1) geometry reconstruction of vertebra and screws, (2) inclusion of metastasis simulating the effect that the metastatic lesion may have in terms of material properties distribution, (3) choice of boundary conditions, and (4) computational discretization of the domain.

**Figure 2:**
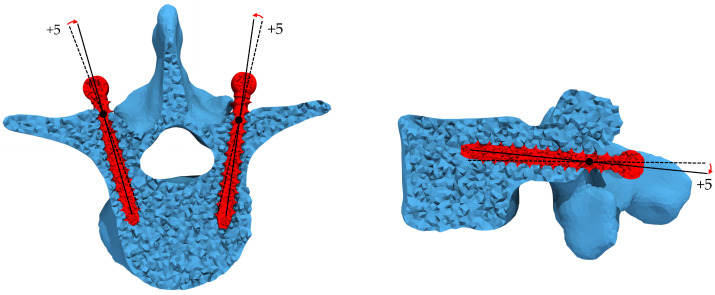
Screw insertion trajectory. Starting from the neutral position (dashed line) corresponding to an angle of 0${}^{\circ }$ in craniocaudal and mediolateral directions, the screws are rotated +5${}^{\circ }$ in both directions (solid line) to obtain craniolateral trajectory.

The vertebral geometry was reconstructed starting from the CT images by segmenting the CT images (ITK-SNAP 3.8.0, University of Pennsylvania, Philadelphia, PA, USA). Then the CAD model of two screws, whose characteristics are reported in [25,26], was generated. For the virtual insertion of the screws within the vertebra, the insertion point was identified by following the clinical indication to simulate a transpedicular trajectory widely adopted during surgical procedures. The screws were inserted at a depth of 30 mm and rotated around the insertion point with an angle of +5${}^{\circ }$ in both craniocaudal and mediolateral directions. As previously mentioned, this specific insertion trajectory was chosen because it resulted in the most critical one, developing high-stress concentrations that may lead to failure of the implant [25,26]. In Figure 2, the specific screws insertion trajectory is shown. However, it is important to emphasize that the screws are not rotated in real surgery. The surgeon identifies the screw insertion point, opens the superficial cortex of the entry point, and inserts a probe to navigate down the isthmus of the pedicle into the vertebral body. The appropriate trajectory in the craniocaudal and mediolateral directions is critical. Finally, the probe is removed, and the pedicle screws are carefully inserted into the same created trajectory. As reported in [25,26], for the virtual insertion, the rotation of screws allows simulating a specific screw trajectory. The bone–screws interface was considered bonded.

Once the screws–vertebra computational domain was reconstructed, it was imported into the Comsol Multiphysics environment (Comsol 5.5, COMSOL, Stockholm, Sweden) for mesh generation. In detail, 10-node tetrahedral elements were used for computational discretization. For the vertebra, the maximum and minimum elements sizes of the mesh were set to 2 mm and 1 mm, respectively, resulting in 212,089 tetrahedral elements and 882,505 nodes, according to a preliminary convergence analysis [25,26]. For the screws, the maximum and minimum elements sizes were set to 1 mm and 0.1 mm, respectively, leading to 62,000 tetrahedral elements. Considering the modified material distribution due to the presence of metastatic lesions, we conducted a further mesh sensitivity analysis obtaining that, for the chosen mesh, the error with respect to an increase in the number of elements of 15% generates a force–displacement curve with a maximum error of about 5% on the computed point. (The reported error was computed as the relative difference between the displacement for a given applied force and the displacement for the finest mesh at the same force, divided by this latter displacement.) Accordingly, the chosen mesh assures a good compromise between accuracy and computational time.

### 2.2. Simulated Metastasis Description

In the Cartesian coordinates system, the mathematical description of the j-th metastasis is expressed by the following relation:

(1) $$g_{j}\left(x,y,z\right)={\left(x-x_{0}\right)}^{2}+{\left(y-y_{0}\right)}^{2}+{\left(z-z_{0}\right)}^{2}-R^{2}=0\;,$$

where $\left(x_{0},y_{0},z_{0}\right)$ and *R* represent the center coordinates and the radius of the j-th metastatic lesion, respectively. The metastasis was simulated within the vertebral domain considering different locations, as will be clarified in Section 2.7, including in all cases, both trabecular and cortical bone.

### 2.3. Constitutive Modeling

The present work’s primary novelty incorporates a specific constitutive description of diseased bone tissue accounting for bone–metastasis interaction in the presence of fixation screws. Following a similar approach previously developed [27,28], we investigated the mechanical behavior of a metastatic femur, overcoming the main limitation of most FE models developed for metastatic vertebra. We assumed an isotropic linearly elastic constitutive law for healthy and pathological bone, though characterized by different material properties. Elastoplasticity was considered only from a numerical point of view, not in a constitutive sense. This was performed through an iterative procedure described in Section 2.6.

Under these assumptions, the stress can be expressed via the second-order Cauchy stress tensor σ as σ=C:ϵ with C the fourth-order elasticity tensor and ϵ the second–order infinitesimal strain tensor. The novelty is based on how C is modeled. In detail, the elasticity tensor C takes into account the effect that metastasis induces in the bone region close to the metastatic lesion, i.e., local degradation of the material properties in agreement with evidence reported in [29]. As a result, the elasticity tensor C is expressed as follows:

(2) $$C\left(x,y,z\right)=C_{CT}\left(x,y,z\right)+\sum_{j=1}^{n}B_{j}\left(x,y,z\right)\;k_{j}\left(x,y,z\right)\left[C_{m}-C_{CT}\left(x,y,z\right)\right]\;,$$

where *n* is the total number of metastases, $C_{CT}\left(x,y,z\right)$ corresponds to the vertebra fourth-order elasticity tensor modeled by assuming the vertebra as heterogeneous and with isotropic constitutive symmetry as explained in the following: $C_{m}$ is the fourth-order elasticity tensor that refers to the isotropic homogeneous linear elastic properties of the metastasis, and $B_{j}\left(x,y,z\right)$ and $k_{j}\left(x,y,z\right)$ are the Heaviside and Gaussian-like functions, respectively, for the j-th metastasis.

Under the assumptions of isotropic heterogeneous linearly elastic behavior, the vertebra elasticity tensor $C_{CT}\left(x,y,z\right)$ is expressed in the function of Young’s modulus *E* and Poisson’s ratio ν. The heterogeneity is related to the distribution of *E* obtained from the CT images. In detail, Young’s modulus for the vertebra was derived by following a procedure that consists of two steps: (1) correction of Hounsfield Unit (HU) values to reduce the partial volume effects [25,26], and (2) conversion of the corrected HU distribution in a heterogeneous distribution of Young’s modulus.

To correct the HU values, the cortical and trabecular regions were first identified based on a representative value of HU equal to 700 (HU≥ 700 identified the cortical region, whereas HU < 700 corresponded to the trabecular region). Then, the HU values in the cortical region were averaged to obtain a mean value of HU ($HU_{mean}$). In the present work, a value of 923 was obtained for $HU_{mean}$. Once the voxels outside of the vertebral domain were identified, the HU value of 923 was assigned to those voxels. Finally, the HU distribution was smoothed by using a moving average filter.

Once the new values of HU were obtained, the HU distribution was converted into a heterogeneous distribution of apparent density ($\rho_{app}$) through the calibration phase obtaining the following relationship:

(3) $$\rho_{app}=1.9\cdot HU/1109$$

with $\rho_{app}$ expressed in g/cm${}^{3}$. For the calibration, since no phantom was included in the CT acquisition, a phantomless approach based on other well-established studies [30,31] was used [25,26]. In detail, a linear correlation among HU and $\rho_{app}$ was assumed, considering that HU = 0 corresponded to an apparent density equal to 0 and the maximum value of HU measured from CT (in this case, HU = 1109) corresponded to an apparent density equal to 1.9 g/cm${}^{3}$ that is the maximum value of $\rho_{app}$ for cortical bone [31]. It is worth noticing that the use of more sophisticated phantomless approaches, such as those reported in [32,33,34], could furnish a more accurate description of vertebra material properties. However, since the aim of the present work is a comparative analysis investigating the effect of different lesion types (i.e., osteolytic and osteoblastic), this approach can be considered acceptable for the present study. Following the calibration, the $\rho_{app}$ distribution was converted in a field of *E* considering two different relationships, one for trabecular bone ($E_{T}$) and the other one for cortical bone ($E_{C}$):

(4a) $$E_{T}=4730\cdot \rho_{app}^{1.56}$$

(4b) $$E_{C}=-892.5\cdot \rho_{app}^{-2.491}+14360$$

with $E_{T}$ and $E_{C}$ expressed in MPa. The relationship in Equation (4a) was developed by Morgan et al. [35], whereas the relationship in Equation (4b) was taken from [25,26].

In terms of Poisson’s ratio ν, a uniform value equal to 0.3 was used for the vertebra [36]. Accordingly, the behavior of the screws was assumed isotropic and linearly elastic, with Young’s modulus and Poisson’s ratio equal to 110 GPa and 0.4, respectively.

Assuming an elastic and isotropic behavior of the metastasis, the elasticity tensor $C_{m}$ results fully described by Young’s modulus and Poisson’s ratio ($E_{m}$ and $\nu_{m}$, respectively) were assumed homogeneous within the metastatic region (but not at the bone–metastasis interface). In detail, for osteolytic metastasis, $E_{m}$ and $\nu_{m}$ were set equal to 0.003 MPa and 0.3, respectively, according to Whyne et al. [11]. For the osteoblastic metastasis, a higher value of $E_{m}$ was used than for the osteolytic lesion. This choice is justified by the evidence reported in [37,38] in which it was reported that osteoblastic metastasis induces unorganized remodeling leading to a large deposition of bone material and seems to be characterized by a high degree of mineralization (i.e., higher stiffness in the FE model). As such, using the maximum CT attenuation value found by Ulano et al. [39] for the osteoblastic lesion (i.e., 787 HU), and computing the $\rho_{app}$ using Equation (3), a value of 14 GPa was obtained for $E_{m}$ through Equation (4b). Poisson’s ratio for the osteoblastic metastasis was set equal to 0.3.

To model the bone–metastasis interaction in terms of degradation of material properties in the bone region close to the metastasis, for the j-th metastasis the Heaviside function $B_{j}\left(x,y,z\right)$, whose value is 1 within the metastasis and 0 otherwise, and the Gaussian-like function $k_{j}\left(x,y,z\right)$ are included in Equation (2) and defined as follows:

(5) $$\begin{array}{ccc} B_{j}\left(x,y,z\right) & = & \left\{\begin{array}{cc} 1 & \;\mathrm{if}\;\;g_{j}\left(x,y,z\right)\leq 0\\ 0 & \;\mathrm{if}\;\;g_{j}\left(x,y,z\right)\;>0 \end{array}\right. \end{array}$$

(6) $$\begin{array}{ccc} k_{j}\left(x,y,z\right) & = & \exp\left[-\tau_{m}\left(\frac{{\left(x-x_{0}\right)}^{2}}{R^{2}}+\frac{{\left(y-y_{0}\right)}^{2}}{R^{2}}+\frac{{\left(z-z_{0}\right)}^{2}}{R^{2}}\right)\right]. \end{array}$$

**Figure 3:**
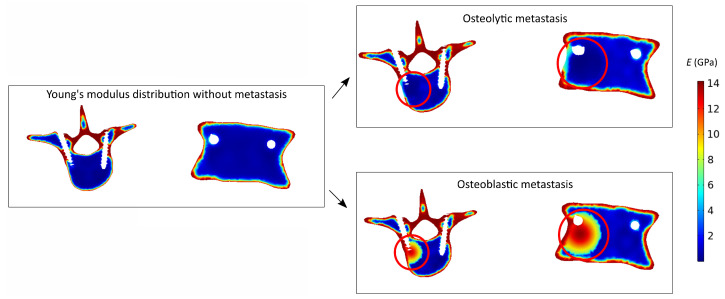
The local effect induced by osteolytic and osteoblastic metastasis on bone material properties is shown. On the left, Young’s modulus *E* distribution without metastasis is reported. On the right, the effects of osteolytic and osteoblastic lesions are circled in red. In the case of an osteolytic lesion, a local weakening is produced, whereas an osteoblastic metastasis induces a local stiffening.

The function $k_{j}\left(x,y,z\right)$ was chosen as a Gaussian-like function calibrated on the center coordinates $\left(x_{0},y_{0},z_{0}\right)$ and radius *R* of the metastatic lesion. This choice was made to induce a local change of Young’s modulus in the bone region close to the metastasis with a smooth transition at the interface between bone and metastasis avoiding sharp constitutive change. As reported in Figure 3, starting from Young’s modulus distribution based on the grayscale of CT images, in the case of an osteoblastic metastasis, such a function induces a local stiffening, whereas in the case of an osteolytic lesion, a local weakening is produced.The term $\tau_{m}$ in Equation (6) is a spatial-degradation-rate parameter that controls the constitutive jump at the bone–metastasis interface. This parameter was set equal to 1 to assign heterogeneous elastic properties at the bone–metastasis interface (about 37%) [27,28] for both types of metastasis. The value chosen for $\tau_{m}$ is in agreement with a study reported in the literature [40] regarding possible degradation features of material properties at the interface between bone and metastasis.

### 2.4. Loading and Boundary Conditions

**Figure 4:**
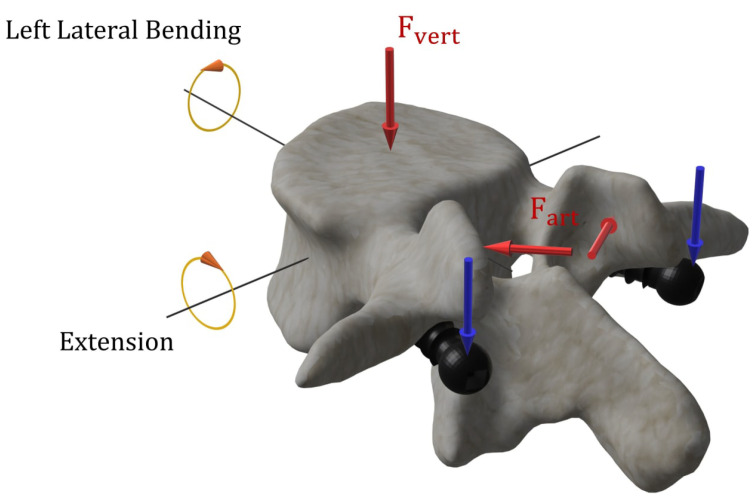
Boundary conditions applied on the vertebra and screws to mimic extension and left lateral bending loading cases. $F_{vert}$ and $F_{art}$ represent the compressive forces applied on the superior end plate and articular facets of the vertebra, respectively. The blue arrows indicate the loading direction applied on the screws. For the two loading cases, a uniform momentum was also applied along two different axes.

Two different loading conditions were simulated in the present work: extension (EXT) and left lateral bending (LLB). To replicate such loading cases, both vertebra and screws were loaded (Figure 4). In detail, 80% of the total load was applied on the vertebra, whereas the remaining 20% was applied on the screws. Regarding the load applied on the vertebra, a compressive force was applied and distributed among the superior end plate ($F_{vert}$ in Figure 4) and the articular facets ($F_{art}$ in Figure 4) of the vertebra. The load on the screws was applied as reported by the blue arrows in Figure 4. In addition, a uniform momentum of 4.7 Nm was applied along the two principal axes for the different tested conditions as shown in Figure 4. For both loading cases, the inferior end plate of the vertebra was fully constrained.

### 2.5. Failure Criteria

We tested two failure criteria to detect the fracture load and the failure patterns of the vertebra: a maximum principal stress criterion and a maximum principal strain criterion.

The maximum principal stress criterion (labeled as $\sigma_{max}$) considers bone completely failed when:

(7a) $$\sigma^{+}>{\bar{\sigma }}^{+}$$

(7b) $$\sigma^{-}>{\bar{\sigma }}^{-}$$

where Equations (7a) and (7b) refer to tensile and compressive failure conditions, respectively. The terms $\sigma^{+}$ and $\sigma^{-}$ correspond to the local measures of stress and are defined as follows:

(8) $$\sigma^{+}=\max\left\{0,\sigma_{1},\sigma_{2},\sigma_{3}\right\}\;,\;\sigma^{-}=-\min\left\{0,\sigma_{1},\sigma_{2},\sigma_{3}\right\}$$

with $\sigma_{1}$, $\sigma_{2}$, and $\sigma_{3}$ the principal stresses; the expressions for the stress limits in tension and compression, ${\bar{\sigma }}^{+}$ and ${\bar{\sigma }}^{-}$, respectively, are defined in terms of ash density ($\rho_{ash}$) as follows [41,42]:

(9a) $${\bar{\sigma }}_{T}^{-}=137\cdot \rho_{ash}^{1.88}$$

(9b) $${\bar{\sigma }}_{C}^{-}=114\cdot \rho_{ash}^{1.72}$$

(9c) $${\bar{\sigma }}^{+}=0.8\cdot {\bar{\sigma }}^{-}$$

where ${\bar{\sigma }}_{T}^{-}$ and ${\bar{\sigma }}_{C}^{-}$ (MPa) are the compressive stress limits for trabecular and cortical bone, respectively, while ${\bar{\sigma }}^{+}$ (MPa) is a unique tensile stress limit. The ash density is derived from $\rho_{app}$ using the following relation [41]:

(10) $$\rho_{ash}=0.551\cdot \rho_{app}-0.00478\;{g/\mathrm{cm}}^{3}$$

The maximum principal strain criterion (labeled as $\epsilon_{max}$) assumes bone failure occurring when:

(11a) $$\epsilon^{+}>{\bar{\epsilon }}^{+}$$

(11b) $$\epsilon^{-}>{\bar{\epsilon }}^{-}$$

with Equations (11a) and (11b) referring to the tensile and compressive failure condition, respectively. The quantities $\epsilon^{+}$ and $\epsilon^{-}$ represent local strain measures, defined in terms of local principal strains ($\epsilon_{1}$, $\epsilon_{2}$ and $\epsilon_{3}$):

(12) $$\epsilon^{+}=\max\left\{0,\epsilon_{1},\epsilon_{2},\epsilon_{3}\right\}\;,\;\epsilon^{-}=-\min\left\{0,\epsilon_{1},\epsilon_{2},\epsilon_{3}\right\}.$$

The terms ${\bar{\epsilon }}^{+}$ and ${\bar{\epsilon }}^{-}$ correspond to density-independent limit strain values in tension (${\bar{\epsilon }}^{+}=0.73\%$) and compression (${\bar{\epsilon }}^{-}=1.04\%$), respectively [43].

For both failure criteria tested, the local failure of the vertebra is assumed to occur when the failure criterion is verified for at least one element. In such occurrence, Young’s modulus of failed elements is set equal to $10^{-6}$ MPa.

### 2.6. Numerical Procedure

A custom Matlab code was developed and integrated within the Comsol Multiphysics environment to simulate the vertebral fracture. The Matlab code and Comsol projects files were uploaded to the open-access repository Zenodo (https://doi.org/10.5281/zenodo.6534415, accessed on 10 May 2022). In detail, a quasi-static force-based incremental approach was adopted to model the progressive damage process. By denoting with $f_{k}$ the force at the *k*-th step, the force at the step *k*+1 ($f_{k+1}$) is expressed as:

(13) $$f_{k+1}=f_{k}+\gamma \Delta f$$

where Δf is the load increment, and it was set equal to 100 N. In the Equation (13), the term γ is a constant that corresponds to a load rate and can be numerically determined as the ratio between *n* and $\bar{n}$$\left(\gamma =n/\bar{n}\right)$, where *n* is the total number of finite elements, and $\bar{n}$ corresponds to the fractured elements at the corresponding step. At each force-based incremental step, the solution is calculated, and the onset of local vertebral failure is verified using the failure criteria described in Section 2.5. If the failure is not locally detected, the applied load is incremented to perform a new numerical step. Otherwise, the material properties of the model are locally modified as described in Section 2.5, and the numerical step is repeated until no further bone failure occurs. The absence of convergence of such a numerical procedure corresponds to a complete failure of the vertebra, i.e., all vertebral elements can be considered failed. In addition to computing stress and strain fields, the vertebral reaction force ($f_{r}$) is computed at each numerical step by performing a numerical integration of the incremental reaction forces over the superior end plate. The corresponding displacement is calculated by averaging the displacements of the nodes at the top end plate. The fracture load ($f_{u}$) is defined as the maximum load computed before an abrupt increase in the top end plate displacement, i.e., a non-converged solution. The fracture pattern is determined by the elements that exhibit failure.

### 2.7. Parametric Analysis

**Figure 5:**
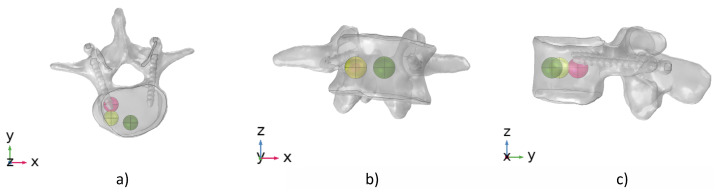
The different positions analyzed for the metastases are shown in transverse (**a**), anterior (**b**), and (**c**) sagittal planes: lateral right P1 (magenta circle); anterior right P2 (yellow circle); and anterior P3 (green circle).

**Figure 6:**
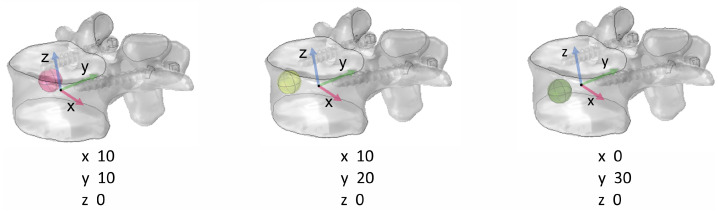
Coordinates of the lesion’s center (in mm) with respect to the center of the vertebral body for each position analyzed. The reference system is localized in the center of the vertebral body. From left to right: lateral right P1 (magenta circle); anterior right P2 (yellow circle); and anterior P3 (green circle).

For each failure criterion (i.e., stress- and strain-based), loading condition (i.e., extension and left lateral bending), and type of metastasis (i.e., osteolytic and osteoblastic), numerical analyses were performed investigating different positions and extensions of a metastatic lesion. In detail, three different locations of the metastasis were considered in agreement with [14,44,45,46,47]: (1) lateral right (P1), (2) anterior right (P2), and (3) anterior (P3) (see Figure 5). To furnish a better understanding of the locations of the metastasis for each position, Figure 6 shows the coordinates of the center of the metastasis with respect to the center of the vertebral body.

Moreover, for each position, the influence of the extension of the metastasis was analyzed. In detail, lesions with a radius of 5 mm ($R_{1}$) and 10 mm ($R_{2}$) were considered [12], corresponding to metastases occupying 2% and 19% of the vertebral body volume, respectively.

Finally, the influence of metastasis shape was investigated for a representative case. This analysis was performed by comparing the presence of one or multiple osteolytic lesions with a radius of 10 mm using the maximum principal strain criterion in the left lateral bending loading condition. Though the single lesion is spherical, the overall metastatic tissue assumes irregular shapes.

A total of 52 numerical analyses were performed (2 loading cases, 2 failure criteria, 2 types of metastasis, 3 different locations, and 2 different radius values). The overall computational time was 7 days on an HP Z640 workstation with E5-2630 v3 (8 × 2.40 GHz) and 32 GB of RAM.

## 3. Results

### 3.1. Fracture Load

A synthetic view of the fracture load values for the complete set of FE analyses is reported in Table 1 and Table 2. In general, for each position and dimension of the metastases, as well as for every loading condition, a strain-based criterion leads to higher values of fracture load compared to those obtained from a stress-based criterion. Such a result is expected due to the sharp edges of the screws leading to a stress concentration phenomenon that reduces the maximum sustainable load. Combining multiple osteolytic lesions to account for the irregular shape of the overall metastatic tissue seems to have a not significant effect on fracture load macroscopically (Table 2).

In the following, the influence of the size, position, shape, and type of metastasis on the mechanical response of the metastatic vertebra is presented and critically discussed.

#### 3.1.1. Size, Position, and Shape Effects

**Figure 7:**
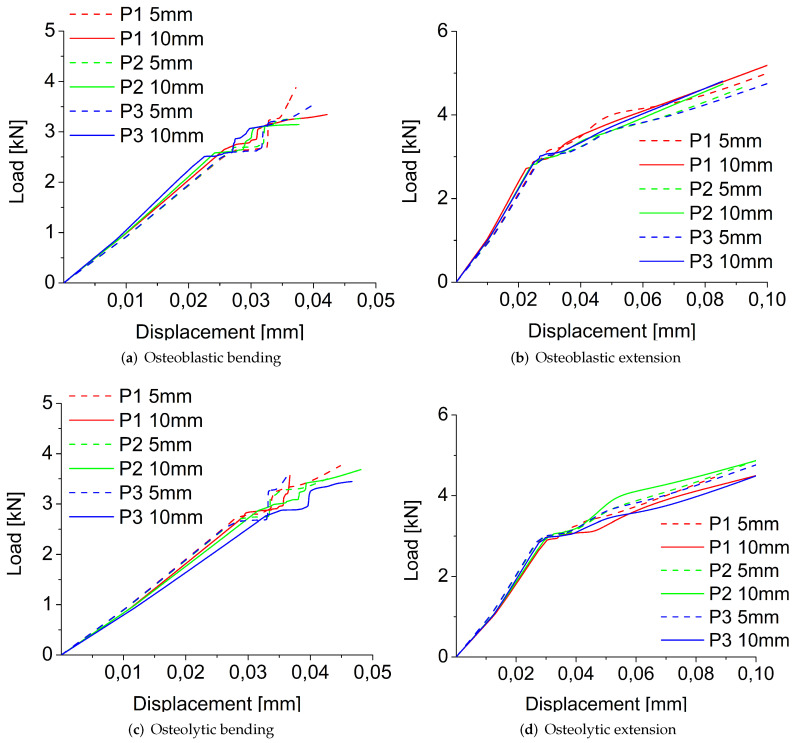
Load-displacement curves obtained for osteoblastic, (**a**,**b**); and osteolytic, (**c**,**d**); metastases in bending, (**a**,**c**); and extension, (**b**,**d**); loading modes using the stress-based failure criterion varying the radius and position of metastasis.

In Figure 7, the load-displacement curves obtained for osteoblastic and osteolytic metastases in bending and extension-loading conditions using the stress-based failure criterion are shown to vary the size and position of the metastasis. In both loading conditions, for all sizes and places, the load-displacement curves of osteolytic and osteoblastic metastases show a linear portion followed by a plastic zone that is wider in extension compared to the bending case (Figure 7).

In bending, for each type of metastasis and position, an increase in lesion radius reduces the fracture load (Figure 7a,c), except for the osteolytic lesion in the P2 position, for which an increase of radius leads to a rise in fracture load (Table 1). In extension, for each position, the increase of the radius of the osteoblastic lesion increases the fracture load as shown in Figure 7b and Table 1. In contrast, in extension for the osteolytic lesion, a greater radius reduces the fracture load in the P1 and P3 positions, whereas for the P2 position, the increase of the metastasis size increases the fracture load (Figure 7d and Table 1).

In bending, for both radii values of osteoblastic metastasis, the fracture load decreases, moving the metastasis from P1 to the P3 position, with the highest fracture load value in the P1 position (closer to the screw) and the lowest value in the P2 position (Figure 7a). In extension, for an osteoblastic lesion of 5 mm of radius, the highest fracture load is detected in the P3 position, whereas the lowest value is in the P1 position (i.e., close to the screw) (Figure 7b and Table 1). Conversely, the fracture load decreases, moving the osteoblastic metastasis with a radius of 10 mm from P1 to P3, with the highest value in P1 and the lowest value in P2 (Figure 7b and Table 1). For the osteolytic metastasis in bending, the fracture load decreases, moving the lesion away from the screw (the lowest fracture load is obtained in the P2 position for metastasis of 5 mm and the P3 position for a metastasis radius of 10 mm) (Figure 7c and Table 1). Conversely, in extension, the fracture load rises if the osteolytic lesion is not close to the screw, with the highest fracture load values in the P3 positions for both radius values. (Figure 7d).

**Figure 8:**
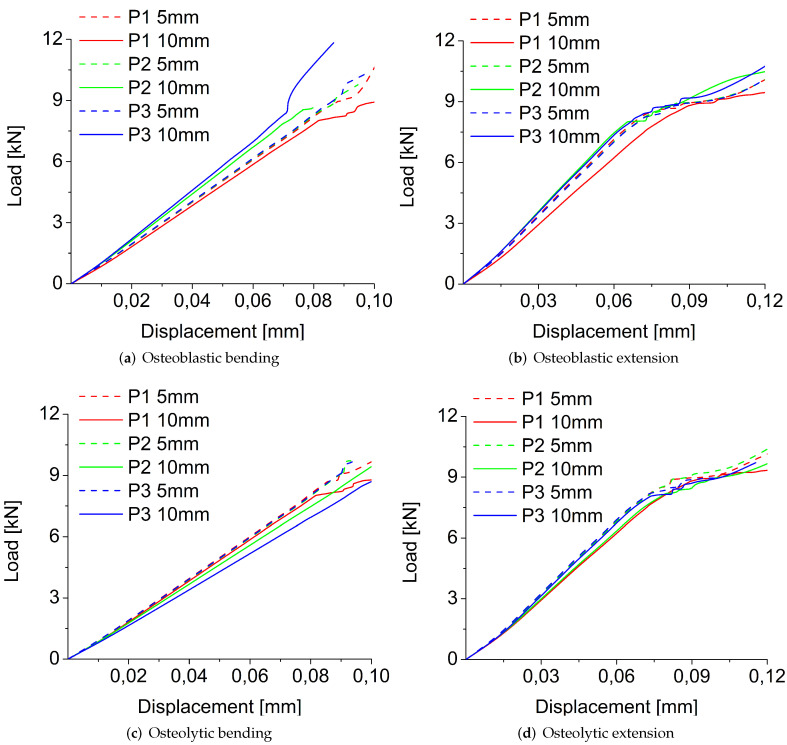
Load-displacement curves obtained for osteoblastic, (**a**,**b**); and osteolytic, (**c**,**d**); metastases in bending, (**a**,**c**); and extension, (**b**,**d**); loading modes using the strain-based failure criterion varying the radius and position of metastasis.

Figure 8 shows the load-displacement curves obtained for osteoblastic and osteolytic metastases in bending and extension-loading conditions using the strain-based failure criterion varying the size and position of the metastasis. In both loading conditions, for all sizes and positions, the load-displacement curves of osteolytic and osteoblastic metastases are characterized by a first linear portion, whereas in extension, the linear part is followed by a plastic zone. This behavior is not observed in bending in which only a linear trend is detected (Figure 8).

In bending for the osteoblastic lesion (Figure 8a), an increase of the metastasis radius leads to an increase of fracture load in P3, but not in P1 and in P2, for which the increase of radius reduces the fracture load (Table 1). On the other hand, for the osteolytic lesion, an increase in metastasis radius decreases the fracture load in all positions (Figure 8c and Table 1). In extension, for the osteoblastic lesion, an increase of radius decreases the fracture load only in P1 but not in P2 and P3, for which an opposite behavior is observed (Figure 8b and Table 1). For the osteolytic lesion, an increase of radius reduces the fracture load in the P1 and P2 positions but not in P3, for which an increase of fracture load is obtained (Figure 8d and Table 1). For the osteolytic and osteoblastic metastases of 5 mm of radius, the highest fracture load value in bending is obtained for the position closer to the screw (i.e., P1). In contrast, the lowest values are detected in P2 and P3 for the osteoblastic and osteolytic, respectively (Figure 8a,c, and Table 1). For a radius of 10 mm, the highest values of fracture load are in P2 and P3 (i.e., in the positions not close to the screw) for osteolytic and osteoblastic lesions, respectively Figure 8a,c, and Table 1). In extension, osteoblastic and osteolytic metastases with a radius of 5 mm are characterized by the lowest fracture load in P3. In contrast, the highest values are in P1 and P2 for the osteoblastic and osteolytic lesions, respectively (Figure 8b,d, and Table 1). On the other hand, both types of metastasis with 10 mm of radius are characterized by the highest fracture load in P3. The lowest values are in P1 and P2 for the osteoblastic and osteolytic lesions, respectively (Figure 8b,d, and Table 1).

**Figure 9:**
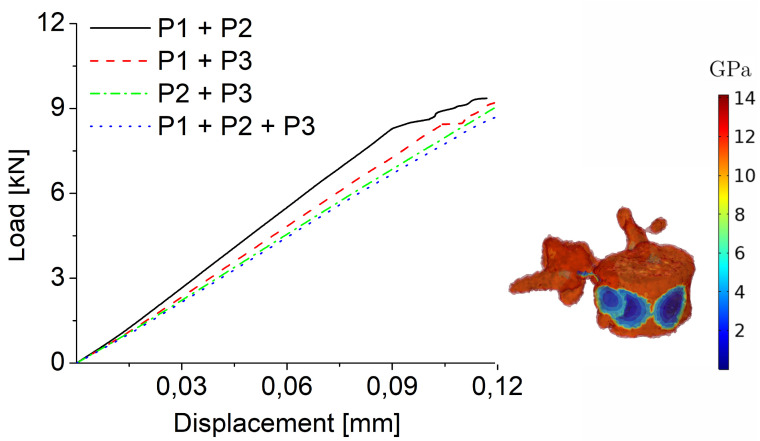
On the left, load-displacement curves obtained for multiple osteolytic lesions with a radius of 10 mm in bending loading mode using the strain-based failure criterion are reported. On the right, the distribution of Young’s modulus in case of multiple osteolytic lesions (P1+P2+P3) is shown.

Finally, comparing the presence of multiple osteolytic lesions, we obtain, as expected, a reduced maximum load (see Figure 9). In particular, the combination of P2 and P3 lesions, both with a radius of 10 mm, represent the most critical scenario, underlying that the position of the lesion (and the associated heterogeneous distribution of material properties) are the prominent effects to consider for predictive vertebra surgery planning.

#### 3.1.2. Comparison between Osteoblastic and Osteolytic Metastases

In most of the analyzed cases, the vertebra with osteoblastic metastasis demonstrated higher fracture loads compared to vertebrae with osteolytic metastasis (Table 1).

**Figure 10:**
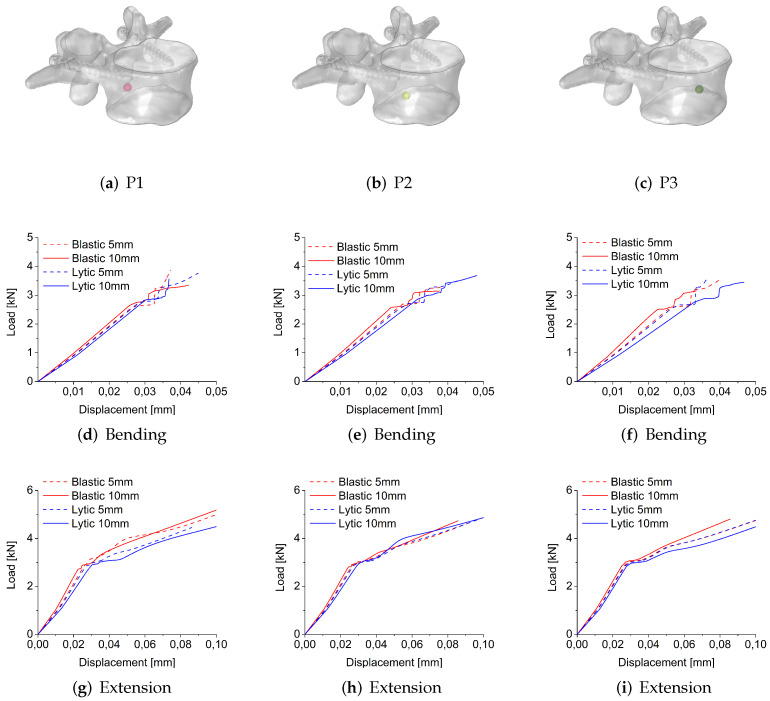
For each position (P1 (**a**); P2 (**b**); P3 (**c**)), the load-displacement curves obtained for osteoblastic and osteolytic metastases with radii of 5 and 10 mm in bending (**d**–**f**) and extension-loading (**g**–**i**) conditions using the stress-based failure criterion are shown.

Figure 10 shows the load-displacement curves obtained for the different positions and radii of lesions in bending and extension-loading modes using the stress-based failure criterion to compare the mechanical behavior of osteoblastic and osteolytic metastases.

In bending, considering the load-displacement curves shown in Figure 10d–f up to a displacement of 0.025 mm (i.e., before the start of fracture propagation), it can be observed that for a radius of 5 mm the osteoblastic and osteolytic metastases seem to have similar behavior with the curves that are overlapped for all the positions. In contrast, for a lesion of 10 mm in all positions, the curves are not overlapped, and a higher stiffness characterizes the osteoblastic lesion than the osteolytic lesion. When the fracture starts to propagate in bending, different trends can be detected between osteoblastic and osteolytic lesions (Figure 10d–f) with differences in fracture load predictions (Table 1). In particular, in the case of a lesion with a radius of 5 mm, the osteoblastic lesion predicts a higher fracture load value than the osteolytic lesion in P1 but not in P2 and P3, where the osteolytic lesion seems to be characterized by a higher fracture load. In contrast, for a lesion of 10 mm of radius, in all positions, the osteolytic lesion predicts a higher fracture load than the osteoblastic lesion.

In extension, the load-displacement curves reported in Figure 10g–i show that overlapped curves characterize osteoblastic and osteolytic lesions with a radius of 5 mm up to a displacement of 0.02 mm (i.e., before the fracture initiation) in all positions, whereas for metastases of 10 mm of radius, a higher stiffness characterizes the osteoblastic lesion compared to the osteolytic lesion, similar to the bending case. When the fracture starts to propagate, by comparing osteoblastic and osteolytic lesions, similar trends of load-displacement curves can be detected in all positions but with differences in fracture load predictions (Figure 10g–i). In detail, in P1 and P2, the osteoblastic lesion of 5 and 10 mm of radius predicts higher fracture load values than the corresponding osteolytic lesions. In P3, the osteolytic and osteoblastic lesions of 5 mm lead to similar values of fracture load, whereas for a lesion of 10 mm of radius, the osteoblastic lesion predicts a higher fracture load than the corresponding osteolytic lesion.

**Figure 11:**
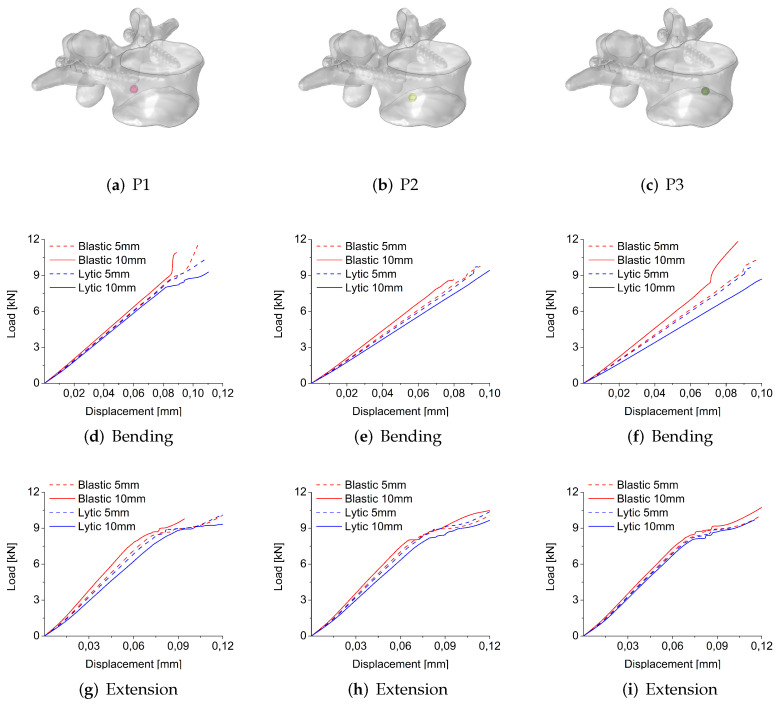
For each position (P1 (**a**); P2 (**b**); P3 (**c**)), the load-displacement curves obtained for osteoblastic and osteolytic metastases of 5 and 10 mm of radius in bending (**d**–**f**) and extension-loading (**g**–**i**) conditions using the strain-based failure criterion are shown.

Figure 11 shows the load-displacement curves obtained for the different positions and radii of the lesion in bending and extension-loading modes using the strain-based failure criterion to compare the mechanical behavior of osteoblastic and osteolytic metastases. In bending, (Figure 11d,e) the load-displacement curves of osteoblastic and osteolytic metastases are characterized by a linear behavior without the plastic zone, highlighting a brittle behavior. The curves are overlapped for osteolytic and osteoblastic lesions of 5 mm in radius. In contrast, for metastasis of 10 mm of radius, the curves reveal the greater stiffness of osteoblastic lesion than the corresponding osteolytic metastasis. From Table 1, it can be observed that in terms of fracture load, in all cases, the osteoblastic lesion predicts a higher fracture load than the corresponding osteolytic metastasis except for a lesion of 10 mm in position P2, for which the osteolytic metastasis indicates the highest fracture load. In extension, the load-displacement curves reported in Figure 11g–i show that osteoblastic and osteolytic lesions with a radius of 5 mm are characterized by overlapped curves up to a displacement of 0.07 mm (i.e., before the fracture initiation) in all positions, whereas for metastases of 10 mm of radius, a higher stiffness characterizes the osteoblastic lesion compared to the osteolytic lesion, similar to the bending case. When the fracture starts, by comparing osteoblastic and osteolytic lesions, similar trends of load-displacement curves can be detected in all positions but with differences in fracture load predictions (Figure 11g–i). In detail, in P1 and P3, an osteoblastic lesion of 5 and 10 mm of radius predicts higher fracture load values than the corresponding osteolytic lesions. In P2, the osteolytic lesion of 5 mm predicts a higher value of fracture load compared to the corresponding osteolytic lesion, whereas for a radius of 10 mm, an opposite situation is observed (Table 1).

### 3.2. Fracture Patterns

In the following, significant examples of the progression of fracture patterns up to the complete rupture of the vertebra are shown.

**Figure 12:**
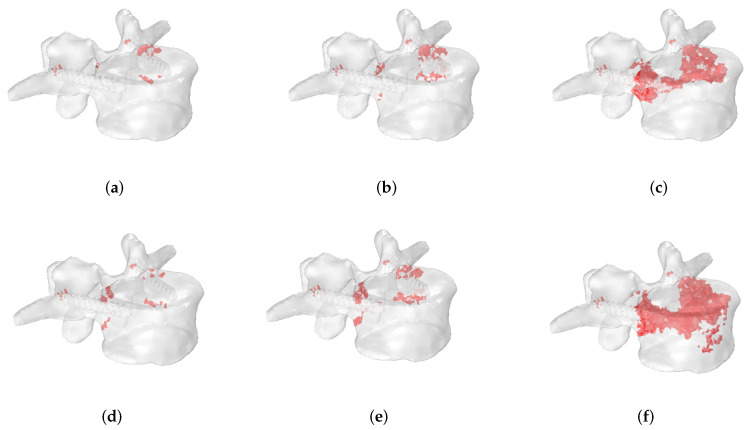
Progression of fracture patterns in an extension-loading condition for osteolytic lesions of 5 mm (**a**–**c**) and 10 mm (**d**–**f**) located in P1 using the stress-based criterion. Failed elements are highlighted in red.

Figure 12 shows the evolution of fracture patterns obtained in extension-loading conditions for osteolytic lesions of 5 mm (Figure 12a–c) and 10 mm of radius (Figure 12d–f) located in P1 using the stress-based criterion. In this case, numerical results demonstrate that a wider metastasis closer to the screw position leads to an extended fracture zone involving a large portion of the vertebral body compared to a smaller lesion.

**Figure 13:**
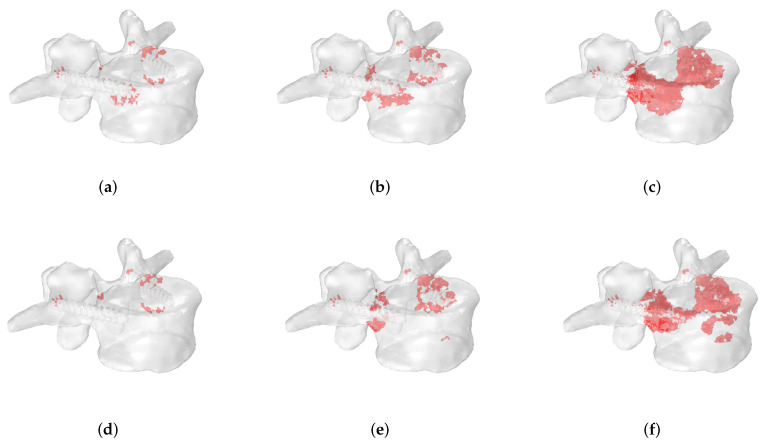
Progression of fracture patterns in an extension-loading condition for an osteoblastic lesion of 10 mm of radius located in P1 (**a**–**c**) and in P3 (**d**–**f**) using the stress-based criterion. Failed elements are highlighted in red.

Figure 13 shows the fracture progression obtained for an osteoblastic metastasis of 10 mm of radius in extension using a stress-based criterion when the lesion is located in the P1 (Figure 13a–c) and P3 (Figure 13d–f) positions. The fracture pattern involves the region close to the screws in both cases. However, failed elements can also be detected when the metastasis is located in P3 (i.e., in the anterior region), demonstrating that the metastasis position with associated heterogeneous material distribution affects the fracture evolution.

**Figure 14:**
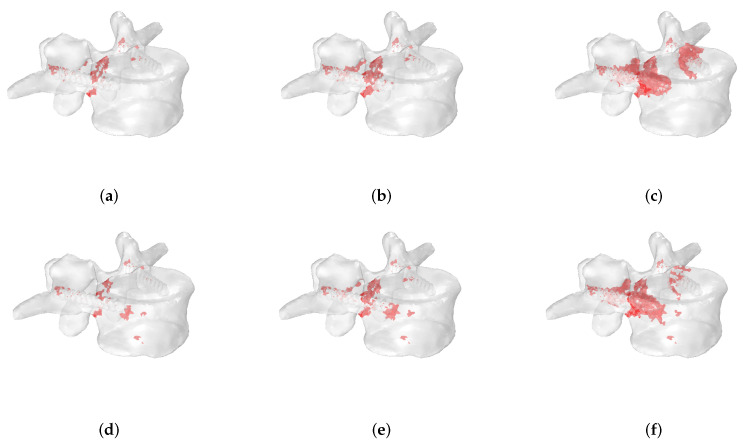
Fracture evolution of an osteolytic (**a**–**c**) and osteoblastic (**d**–**f**) lesion of 10 mm of radius located in P2 in a bending loading condition using a stress-based criterion. Failed elements are highlighted in red color.

Figure 14 compares the fracture evolution between osteolytic (Figure 14a–c) and osteoblastic (Figure 14d–f) lesions, considering a lesion of a radius of 10 mm, located in P2 and undergoing bending loading. A stress-based criterion is reported, whereas the osteolytic metastasis shows a fracture pattern that involves both screws, and the osteoblastic lesion presents failed elements concentrated closer to the screw and located on the same side of the metastasis. This also suggests that the type of metastasis can influence the fracture pattern.

## 4. Discussion

The present study aimed to investigate the mechanical behavior of instrumented metastatic vertebra by developing an image-based FE computational approach that accounts for the bone–metastasis interaction in terms of local change of material properties within the metastatic lesion. The study was designed through an extended FE parametric analysis to assess the influence of type, size, location, and shape of metastasis on the mechanical response of the vertebra, comparing fracture load and fracture patterns.

Numerical results demonstrate that the size of metastasis affects the prediction of fracture load of the vertebra with a different impact based on the type of metastasis. The increasing size of osteolytic lesions reduces the fracture load in almost all investigated cases. This result is in agreement with [14] in which the authors found a strong linear relationship between the increase of the size of the osteolytic lesion and the decrease in ultimate force. The lowering in fracture load can be explained by the reduction in stiffness that involves a greater portion of the vertebra when the size of metastasis increases. In contrast, for the osteoblastic case, a non-univocal effect is detected, and it seems to be dependent on loading condition. Whereas in bending, a larger size of osteoblastic lesion leads to a lower fracture load, in extension, an opposite behavior is obtained. Such an undefined behavior is in agreement with Palanca et al. [48], showing that the vertebra with osteolytic lesion exhibited more critical deformations in all cases compared to the non-metastatic vertebra. In contrast, a vertebra with an osteoblastic lesion showed deformations similar to or lower than the non-metastatic cases.

The numerical results obtained in the present study demonstrated that the location of the metastasis could influence the overall mechanical response of a diseased vertebra, in agreement with previous studies emphasizing a non-negligible role of the metastasis location in predicting the vertebral strength [13,48]. However, it is worth noting that in [14], a non-clear effect of the location of lytic lesions on predicted structural properties was reported.

In this contribution, a metastasis located closer to the screw leads to a higher fracture load than a metastasis situated far from the screw in the majority of the cases. This result can be justified by the higher stiffness of the screw that induces stress concentration in the surrounding tissue. In addition, the loading mode is crucial in determining the fracture pattern in combination with the position effect. In some extension-loading conditions, it was found that the fracture load is higher far from the screw. This result highlights the importance of patient-specific models necessary for a definite and personalized clinical assessment. The type of metastasis has an impact on fracture load prediction, with higher fracture loads predicted for osteoblastic lesions (Table 1). This result is in agreement with findings reported in [10] indicating the need for multiscale-material-modeling approaches based on mechano-biological assumptions for the correct modeling of altered bone tissue. The negligible influence of the irregular shape of overall metastatic tissue on fracture load seems in agreement with a few studies reported in the literature [12,49]. However, this aspect requires a deeper investigation to evaluate the effect of combining the irregular shape with stress-based criteria, osteoblastic lesions, and multiple loading conditions.

An additional result of our computational approach comes from the visual inspection of the fracture pattern distribution within the vertebra body. Such a feature is of crucial importance from a clinical point of view, indicating and quantifying the location and distribution of the damaged volumes in view of an optimized surgery procedure.

The adopted constitutive modeling strategy moves toward defining a refined representation of metastatic lesions. In some studies, the metastatic tissue is modeled as void. However, such an approach neglects specific material properties of diseased tissues as well as the biomechanical interaction between the lesion and the surrounding environment. On the other hand, another widely adopted approach consists of modeling metastasis as healthy bone. Again, the tumor environment features altered constitutive parameters and requires advanced modeling strategies [50]. Therefore, there is an urgent need to develop suitable material models for describing metastatic tissues [51,52,53,54]. In the authors’ opinion, accounting for specific material properties for the metastasis and thus developing a more accurate constitutive description of the diseased tissue can furnish a straight indication of stress–strain local stimuli within the metastatic tissue and at the bone–metastasis interfaces. In the framework of multiscale modeling strategies, this aspect is crucial for identifying mechano-biological effects regulating disease evolution and remodeling processes, thus predicting tumor growth. Considering that most pathological fractures of metastatic vertebrae are related to tumor progression, the results discussed in the present study lay down a robust computational framework for preventing fracture events in silico. Moreover, accurate descriptions of tissue morphology and properties in the presence of metastasis have great importance for developing effective tumor-drug transport models [55,56,57], and thus defining patient-specific effective therapeutic strategies.

### Limitations and Perspectives

We point out limitations and future perspectives of our study. Starting from the constitutive description, bone and metastasis were considered isotropic linear elastic materials. As already mentioned, the tumor mechanical microenvironment is characterized by a porous solid matrix filled with interstitial fluids. Accordingly, a poroelastic constitutive description may be considered as a generalized modeling approach to improve our understanding of the overall mechanical behavior of metastatic vertebrae [27,28]. Furthermore, integrating an accurate multiscale description of the tumor microenvironment would help detect local stimuli which are critical for identifying mechano-biological pathways regulating disease evolution and remodeling processes [58]. In fact, anisotropy characterizes vertebra trabecular bones. Even if the isotropy can be considered a reasonable assumption in the present work—due to its comparative character—the inclusion of patient-specific anisotropy (e.g., accounting for image-based methods as gray-level structure tensors) is expected to enhance the reliability of the proposed computational approach.

Considering modeling parameters, the shape of the function $k_{j}$ and the values of $\tau_{m}$ adopted in the present work represent a possible general choice. However, due to the lack of experimental evidence, these functions can be considered the first attempt to investigate the effect of bone degradation induced by a metastatic lesion. Proposed results, though not conclusive, represent a rigorous contribution toward the proper definition of effective modeling strategies and formulations in this context. Similarly, the phantomless approach used can be considered a first approximation for obtaining patient-specific material properties distribution of the vertebra. As such, sophisticated methods [32,33,34], as well as the use of calibration phantoms during CT acquisition, could furnish a more accurate description of local constitutive properties. Here, dedicated experimental measurements on metastatic tissues are mandatory to identify the most reliable material parameters within an intrinsic biological variability.

From a numerical and statistical viewpoint, the parametric analysis was conducted on a single patient. Thus, the study should be extended to a broader clinical cohort to corroborate and generalize our results. Moreover, the presence of mixed metastasis (i.e., metastasis characterized by a mix of lytic and blastic features) should be investigated as in [48]. Finally, studying the mechanical behavior of metastatic vertebrae in which bone cement augmentation and screw fixation are applied together could be another factor to consider. Recently, it was reported that both techniques can be used together to stabilize metastatic vertebrae [59,60].

It is worth noting that the use of clinical datasets is intrinsically affected by the lack of experimental data to validate numerical findings. Considering the primary purpose of the present study, i.e., to provide a rigorous and reliable computational method to predict in silico the complex fracture patterns occurring in metastatic vertebrae, we hope that extensive experimental validation is made available in the near future to corroborate and augment our results.

## 5. Conclusions

We presented a new image-based computational FE approach accounting for the effect of metastasis on bone material properties. A numerical tool was developed and used to evaluate the impact of size, location, and type of metastasis on the mechanical response of an instrumented human vertebra. Our analysis highlighted that these parameters significantly affect fracture load predictions and patterns. Moreover, improved modeling of the bone–metastasis interaction plays a crucial role in assessing the mechanical behavior of metastatic vertebra. Our study moves toward the definition of robust and reliable computational tools expected to be used by clinicians to improve the management of metastatic vertebrae. In particular, integrating numerical tools to predict complex fracture mechanisms would certainly help an optimal surgical treatment plan. The final objective is to minimize the risk of fracture and, thus, implant failure to improve the management of patients with metastatic disease.

## Figures and Tables

**Table 1:** The fracture load (FL) expressed in N is reported for each loading condition, failure criterion, type, size, and location of metastasis. EXT (extension) and LLB (left lateral bending) correspond to the loading cases analyzed. The symbols $\sigma_{max}$ and $\epsilon_{max}$ refer to maximum principal stress and strain criteria, respectively. P1 (lateral right), P2 (anterior right), and P3 (anterior) correspond to locations of metastases. $R_{1}$ and $R_{2}$ are the radii of metastasis (5 and 10 mm, respectively).

Metastasis	Radius	EXT						LLB					
		$\sigma_{\max}$			$\epsilon_{\max}$			$\sigma_{\max}$			$\epsilon_{\max}$		
		**P1**	**P2**	**P3**	**P1**	**P2**	**P3**	**P1**	**P2**	**P3**	**P1**	**P2**	**P3**
		**FL**	**FL**	**FL**	**FL**	**FL**	**FL**	**FL**	**FL**	**FL**	**FL**	**FL**	**FL**
Osteoblastic	$R_{1}$	4467	4709	4751	10,388	9775	9602	3877	3269	3543	11,704	9773	10,368
	$R_{2}$	5570	4741	4809	9781	10,402	11,905	3343	3145	3148	10,907	8632	11,829
Osteolytic	$R_{1}$	4456	4111	4788	10,138	10,811	8947	3763	3508	3581	10,466	9713	9678
	$R_{2}$	4045	4253	4659	9449	9402	9711	3565	3685	3446	9284	9645	8937

**Table 2:** The fracture load (FL) expressed in N is reported for the cases in which multiple osteolytic lesions are considered. Results are reported in left lateral bending loading condition and using the maximum principal strain criterion.

Metastasis	Radius	LLB			
		$\epsilon_{\max}$			
		**P1+P2**	**P1+P3**	**P2+P3**	**P1+P2+P3**
		**FL**	**FL**	**FL**	**FL**
Osteolytic	$R_{2}$	9359	9362	9373	9024
